# Bevacizumab dose adjustment to improve clinical outcomes of glioblastoma

**DOI:** 10.1186/s12916-020-01610-0

**Published:** 2020-06-22

**Authors:** N. García-Romero, I. Palacín-Aliana, R. Madurga, J. Carrión-Navarro, S. Esteban-Rubio, B. Jiménez, A. Collazo, F. Pérez-Rodríguez, A. Ortiz de Mendivil, C. Fernández-Carballal, S. García-Duque, J. Diamantopoulos-Fernández, C. Belda-Iniesta, R. Prat-Acín, P. Sánchez-Gómez, E. Calvo, A. Ayuso-Sacido

**Affiliations:** 1 Fundación de Investigación HM Hospitales, HM Hospitales, Madrid, Spain; 2 Faculty of Experimental Sciences, Universidad Francisco de Vitoria, Pozuelo de Alarcón, Madrid, 28223 Spain; 3 Atrys Health, Barcelona, 08025 Spain; 4 Fundación Vithas, Vithas Hospitals, Madrid, 28043 Spain; 5 Formerly: Fundación de Investigación HM Hospitales, HM Hospitales, Madrid, Spain; 6 Formerly: Facultad de Medicina (IMMA), Universidad San Pablo-CEU, Madrid, Spain; 7 Servicio de Neurocirugía, Hospital General Universitario Gregorio Marañón, Madrid, Spain; 8 Departamento de Neurocirugía, Hospital Universitario la Fe, Valencia, Spain; 9 Neuro-oncology Unit, Instituto de Salud Carlos III-UFIEC, Madrid, Spain; 10 START Madrid-CIOCC, Centro Integral Oncológico Clara Campal, Madrid, Spain; 11 Formerly: Facultad de Medicina (IMMA), Universidad San Pablo-CEU, Madrid, Spain

**Keywords:** VEGFA, Angiogenesis, Bevacizumab, Glioblastoma, Neovasculogenesis

## Abstract

**Background:**

Glioblastoma (GBM) is one of the most aggressive and vascularized brain tumors in adults, with a median survival of 20.9 months. In newly diagnosed and recurrent GBM, bevacizumab demonstrated an increase in progression-free survival, but not in overall survival.

**Methods:**

We conducted an in silico analysis of VEGF expression, in a cohort of 1082 glioma patients. Then, to determine whether appropriate bevacizumab dose adjustment could increase the anti-angiogenic response, we used in vitro and in vivo GBM models. Additionally, we analyzed VEGFA expression in tissue, serum, and plasma in a cohort of GBM patients before and during bevacizumab treatment.

**Results:**

We identified that 20% of primary GBM did not express VEGFA suggesting that these patients would probably not respond to bevacizumab therapy as we proved in vitro and in vivo*.* We found that a specific dose of bevacizumab calculated based on VEGFA expression levels increases the response to treatment in cell culture and serum samples from mice bearing GBM tumors. Additionally, in a cohort of GBM patients, we observed a correlation of VEGFA levels in serum, but not in plasma, with bevacizumab treatment performance.

**Conclusions:**

Our data suggest that bevacizumab dose adjustment could improve clinical outcomes in Glioblastoma treatment.

## Background

Glioblastoma (GBM), a grade IV glioma according to the World Health Organization (WHO) classification, is one of the most common, aggressive, and highly vascularized brain tumors in adults, with a median survival of 20.9 months and an incidence rate of 3–4 newly diagnosed patients per 100,000 population [1–3]. The gold standard treatment consists of a maximal surgical resection, followed by radiotherapy with concomitant and adjuvant chemotherapy with temozolomide [4]. Recently, it has been observed that using tumor-treating field (TTField) maintenance in combination with temozolomide has improved GBM overall survival (OS) with minor side effects [3]. However, despite advances in surgical techniques and efforts in the development of new drugs, increasing patient survival rates is still a challenge.

GBMs are characterized by their low tumor cell differentiation, high proliferation and intracranial dissemination, elevated cellular heterogeneity, and abundant normal and aberrant vasculature [1, 5]. Pathological neovascularization is mostly triggered by hypoxia through the expression of vascular endothelial growth factor (VEGF) [6], which promotes angiogenesis, vasculogenesis, and vascular mimicry, cellular and molecular mechanisms that usually overlap each other [7].

Anti-angiogenic therapies have been shown to normalize the structure and function of tumor-associated blood vessels [8]. Bevacizumab (Avastin®, Genentech/Roche), a recombinant humanized IgG1 monoclonal antibody that prevents the interaction of VEGFA with its receptors and inhibits downstream signaling pathways [9], was the first anti-angiogenic immunotherapy agent approved by the FDA to be used in GBM and in other cancer pathologies [10]. Bevacizumab blocks the autocrine and paracrine VEGF signaling and reduces vessel leakiness and pressures within the brain tumor, triggering a vasogenic edema reduction and, consequently, lowering corticosteroid administration [11, 12]. Additionally, it may lead to vessel regression, thus depriving cancers of their nutrient source, which in turn may synergize the response to chemotherapy [13, 14]. However, some phase II and phase III clinical trials performed with bevacizumab in newly diagnosed (AVAglio-B021990 and RTOG-0825) and recurrent GBM patients failed to demonstrate a significant OS advantage, while revealing strong evidence of prolonged progression-free survival in GBM [15–19]. Indeed, the same results were observed in a recent meta-analysis carried out by Cochrane, in which authors emphasize that none of the clinical assays address the possibility that subsets of patients with GBM may benefit from bevacizumab therapy [20]. Additionally, some GBM tumors grow in an angiogenesis-independent manner suggesting that these patients would probably respond poorly to anti-angiogenic therapy. Therefore, it is essential to identify a patient subpopulation that will benefit from the bevacizumab treatment [21]. Although some predictive biomarkers have been suggested to be measured in plasma [22] and in solid biopsies [23], more robust and efficient biomarkers are needed to identify responder and non-responder patient subpopulations as well as to personalize the bevacizumab doses required to achieve antitumor effectiveness in the responder group.

We found that VEGFA expression levels could be used to calculate the specific dose of bevacizumab needed to increase the response to bevacizumab treatment. Additionally, GBM cell lines and patients with initial high VEGFA levels were more likely to respond positively to this anti-angiogenic therapy than other subpopulations of GBM with low VEGFA expression-secretion levels. Moreover, in a small cohort of GBM patients, we observed that VEGFA levels in serum, but not in plasma, correlated with response to bevacizumab treatment. Our data suggest that analyzing VEGFA levels in serum could be useful as a predictive biomarker to identify GBM patients that may benefit from bevacizumab treatment as well as to guide the recommended dosage.

## Methods

### Cell lines and culture

Three immortalized GBM cell lines (LN229, U87, and U373), human brain microvascular endothelial cells (HBMECs), and human mesenchymal stem cells (hMSCs) (a gift from Dr. Carmen Escobedo Lucea) were used. These cell lines were cultured in Dulbecco’s modified Eagles’ medium (DMEM) supplemented with 10% fetal bovine serum and 100 units/ml penicillin and 100 mg/ml streptomycin and were cultured in parallel at normoxia (21% O_2_) and hypoxia (1% O_2_). The GBM 12 OCT primary line was provided by Pilar Sanchez Gómez (Instituto de Salud Carlos III-UFIEC) [24], and HBMECs were isolated following the protocol published by Stins and colleagues [25].

Cancer stem cells (GBM27, GBM123, GBM128D, GBM38, GBM128B, and GBM18) were isolated from GBM surgical samples and cultured according to the protocol described previously [26]. Tissue samples were obtained from patients operated at the Neurosurgery Department of Hospital Universitario la Fe (Valencia, Spain).

### Bevacizumab treatment in vitro

Cells were grown for 72 h, and levels of VEGFA basal secretion were calculated by enzyme-linked immunosorbent assay (ELISA). Then, the amount of bevacizumab (Avastin®, Roche) needed to neutralize the VEGFA secretion was calculated in each case (Additional file: material and methods section).

### Enzyme-linked immunosorbent assay (ELISA)

Basal levels of VEGFA in vitro or total serum levels isolated from mice and humans were calculated by ELISA, according to the VEGF Human ELISA Kit manufacturer’s instructions (Invitrogen). Absorbance detection was performed at 450 nm using a microplate reader.

### RT and QRT-PCR

For RT-PCR, total RNA was isolated using RNeasy Mini or Micro kit (QIAGEN) following the manufacturer’s recommendations. One microgram of RNA was used for cDNA synthesis (High-Capacity cDNA Reverse Transcription Kit; Applied BioSystems). QRT-PCR reactions were performed in an optical 384-well plate equipped with an ABI PRISM 7900 HT sequence detection system (Applied Biosystems) using SYBR Green. Gene expression levels were quantified using the primers listed in Table 1, and two housekeeping genes were used to normalize the data.

**Table 1:** Primers used to quantify gene expression levels

	Forward 5′→3′	Reverse 5′→3′
Human primers		
*VEGFA*	CTAACACTCAGCTCTGCCC	ACACACAAATACAAGTTGCCAA
*GAPDH*	TCCTCCACCTTTGACGCTG	ACCACCCTGTTGCTGTAGCC
*β-actin*	CATCCCCCAAAGTTCACAAT	ATGGCAAGGGACTTCCTGTA
*β-2 microglobulin*	TTCTACTTTGAGTGCTGTCTCCA	ATTCTCTGCTCCCCACCTCT
Mouse primers		
*VEGFA*	ACACGGTGGTGGAAGAAGAG	GGAAGGGAAGATGAGGAAGG
*VEGF-B*	TATCTCCCAGAGCTGCCATC	CCCAAATCCCGTTATTGGTA
*VEGFR-2*	GGCAGTGTCTGAGGGTTCTC	TGGAGAGCAAACCAACCAAT
*CD31*	CGGTTTCCTAAGGTCTGAGC	GAGAAGGCGAGGAGGGTTAG
*vWF*	CAATGGCTCTGTCGTGTACC	CAAGGGAGGGATCTGGTTTT
*GAPDH*	TGGCATCCTTGCTTACACAG	GCAATTCCAGCCTTAGCATC
*β-actin*	AATTTCTGAATGGCCCAGGT	GCTGCCTCAACACCTCAAC
*β-2 microglobulin*	GCACGCAGAAAGAAATAGCA	CAGAGGGTTTGGCATATGAT

### Protein isolation and Western blotting

Cells and tumor specimens were lysed with radioimmunoprecipitation assay buffer (RIPA) containing a protease and phosphatase inhibitor cocktail (Roche). After centrifugation, the supernatant was collected and quantified using the BCA Protein Assay Kit (Calbiochem). Protein extracts were then separated by 12% SDS-PAGE and transferred to nitrocellulose membranes. After blocking for 1 h with 10% bovine serum albumin in T-TBS, the membranes were incubated with primary antibody against VEGFA (ab46154) overnight at 4 °C. After washing, the membranes were incubated with the corresponding peroxidase-labeled secondary antibody. Detection was performed using ECL reagents according to the manufacturer’s guidelines. Band intensities were quantified using Quantity One software (Bio-Rad Laboratories, Inc.) and were standardized to β-actin or GAPDH levels.

### Immunofluorescence staining

Cells were cultured in chamber slides and then fixed with 4% paraformaldehyde. After blocking for 1 h at room temperature (RT) with 10% BSA and 0.05% Triton X-100 in PBS, staining was performed using rabbit monoclonal anti-VEGFA (1:200, ab52917) followed by overnight incubation at 4 °C. Slides were washed and incubated for 1 h at RT with secondary antibody Alexa 555 goat anti-rabbit (1:200, Invitrogen) and Alexa Fluor 647 Phalloidin (1:100, Invitrogen). Then, the nuclei were stained with DAPI, (1:5000, Sigma-Aldrich), and slides were mounted with fluorescence mounting medium FluorSave™ reagent (Millipore). Fluorescence was observed under a Leica TCS SPE-inverted confocal microscope in the Confocal Unit of IiSGM. The experiment was performed twice, and in each event, three images of the different types of cells were taken. Negative controls with the secondary antibodies were carried out in all cases.

Tumor tissue sections were immersed in a 5% blocking solution of the specific serum and then incubated (overnight, 4 °C) in solutions containing the following primary antibodies: goat anti-mouse CD105 (R&D Systems) and mouse anti-human vimentin (Santa Cruz Biotechnology). Then, Alexa Fluor-conjugated secondary antibodies were used for 1 h (donkey anti-goat 555 and goat anti-mouse 488), and then the nuclei were counterstained with DAPI and coverslips were mounted using FluorSave™ reagent (Millipore). The immunofluorescence results were quantified using the Image-Pro Plus software where the fluorescence intensity of CD105 and VEGFA was calculated.

### Conditioned media

To produce conditioned media (CM), LN229, U87, and U373 were cultured for 72 h in the presence of IgG and standard or specific doses of bevacizumab. CM was sterile-filtered and stored at − 80 °C until use.

### Tube formation

Matrigel™ Matrix High Concentration (BD Biosciences) was diluted with DMEM and used to coat 96-well plates at 37 °C for 30 min. Then, 2 × 10^4^ HBMECs were resuspended in conditioned media DMEM 1:1 and seeded onto the Matrigel. The tube formation ability was measured after O/N incubation. The total length of the tubes and the number of branches were quantified using ImageJ.

### Transwell migration assay

To evaluate cell migration, a 24-well plate with 8-μm pore size polycarbonate membrane inserts was used. Approximately 7.5 × 10^4^ HBMECs were resuspended in 100 μl serum-free DMEM medium and seeded in the upper chamber, and 500 μl of conditioned media was added to the lower chamber. After 3 h, the migrated cells were fixed with 4% paraformaldehyde and stained with 0.1% crystal violet. All experiments were performed in triplicate.

### Wound healing assay

HBMECs were seeded in a 24-well plate overnight. The medium was removed, and the cells were washed twice with DMEM. A linear wound was created dragging a 200-μl pipette tip across the surface. Conditioned media from U87, U373, and LN229 cell lines with and without standard and specific doses of bevacizumab were added. The wound area was photographed with phase contrast at 0 h, and cells were allowed to migrate for 7 h. Scratched areas were measured with the ImageJ software, and the percent of wound closure was calculated.

### xCELLigence real-time cell analysis (RTCA): migration

4 × 10^4^ HBMECs were seeded per well into E16 plates (Roche) in DMEM, and conditioned media were loaded to the lower chamber. Then, the E16 plates were equilibrated to 37 °C and placed into the xCELLigence system. The impedance was recorded in 15 min intervals for 72 h, and the total number of cells migrating was quantified using a cell index.

### In vivo Matrigel plug assay

Conditioned media of each cell line were concentrated using Amicon Ultra-15 (Millipore). Then, 150 μl of media concentrate was mixed with 350 μl Matrigel (BD Bioscience) and heparin. The mixture was injected subcutaneously into the flanks of 6-week-old immunodeficient mice (*N* = 5 mice per group). Mice were sacrificed after 7 days, and Matrigel plugs were removed.

### Histopathology

Matrigel plugs and tumor tissues were fixed in 4% paraformaldehyde and embedded in paraffin, and 4-μm slices were obtained. The slides were stained with hematoxylin and eosin (H&E). Masson Trichrome Goldner staining was performed according to the manufacturer’s instructions (Bio Optica).

### Heterotopic xenograft assay and bevacizumab treatment

1.5 × 10^6^ LN229, U87, and U373 cells were resuspended in culture media, mixed with cold liquid Matrigel (BD), and then subcutaneously injected into athymic nude mice (*N* = 10 per group). Following Baumgarten’s recommendations for dose conversion between human/iv and mice/ip [27], specific and standard doses were calculated using in vitro hypoxic basal secretion. Final concentrations used were as follows: 60 mg/kg (U373-specific dose), 34 mg/kg (U87-specific dose), 10 mg/kg (LN229-specific dose), and 16.6 mg/kg bodyweight (standard dose). A control group (*N* = 10) received human polyclonal immunoglobulin (IgG, Intratect) at the same concentration as bevacizumab. Tumor volumes were measured with a caliper when they reached a visible size, and mice were euthanized when tumor burden became symptomatic.

### Tissue microarray (TMA)

Eighty-seven tissue blocks were evaluated by a pathologist for the presence of malignant sections. Tumors were graded following the World Health Organization classification and divided into LGG and GBM (Additional file 1: Tables S1-2). Six TMAs were constructed from those formalin-fixed paraffin-embedded (FFPE) tissues using an arraying instrument (GALILEO CK 3500). Three tissue cores (0.6–1 mm diameter each) were made from each tissue block. Then, TMA blocks were cut at 4 μm and stained with hematoxylin & eosin. VEGFA signal was scored as follows: 0 = rare, 1 = localized, and 2 = diffuse.

### Immunohistochemistry

FFPE and TMA sections were stained (as per the manufacturer’s staining protocol) with the Bond Polymer Refine Detection Kit on a Bond-max™ fully automated staining system (Leica Microsystems GmbH, Germany), using a rabbit polyclonal antibody against VEGFA (1:100, ab46154).

### Human samples

Solid surgical tissue samples and peripheral blood from patients and healthy donors were obtained from patients operated at HM Hospitales (HM), Madrid, Spain; Biobanco La Fe (PT17/0015/0043), Hospital Universitario la Fe (HUlaFe), Valencia, Spain; and the HGM BioBank, integrated in RETICS, National Network Biobanks, funding by Instituto de Salud Carlos III (Additional file 1: Tables S1-2). Peripheral blood samples from patients were collected prior to surgery, or prior and during Avastin treatment. These blood samples were left to clot for 30 min at room temperature, and the serum was isolated and stored at − 80 °C until use.

### MRI

MR images were obtained using a 3-T MRI scanner (Achieva Intera 3T; Philips Healthcare, Best, the Netherlands) and 8-channel SENSE head coil. For DCE-MRI, baseline T1-weighted images were obtained with the following parameters: TR 3.5 ms, TE 1.7 ms, slice thickness 3.5 mm, field of view (FOV) 230 mm, matrix size of 116 × 117, 30 volumes, temporal resolution 5.7 s, and flip angles of 5° and 10° to create two pre-contrast datasets. Then, a DCE perfusion imaging dynamic series was performed using T1-weighted sequences with the same MR parameters except for an increased flip angle of 10°. At the end of the third volume acquisition, a bolus of 3 ml of gadolinium (Gd)-based contrast agent (gadobutrol 1 mmol/ml) was injected intravenously at a rate of 3 ml/s.

Structural contrast 3D T1 fast field echo (FFE) sequence was then performed, and the detail parameters were as follows: TR/TE = 5.8/2.7 ms, flip angle 8°, FOV 240 × 192 mm, matrix size 240 × 192, and reconstructed voxel size of 1 × 1 × 1 mm.

At that point, a T2-weighted perfusion MRI was performed. At the end of the third volume acquisition, a bolus of 6 ml of gadolinium was injected intravenously at a rate of 5 ml/s. The sequence was done with the following parameters: TR 17 ms, TE 25 ms, slice thickness 3.5 mm, field of view (FOV) 230 × 184 mm, matrix size of 80 × 60, 30 volumes, temporal resolution 2.6 s, and flip angles of 7°.

Parametric maps of cerebral blood volume (CBV) were acquired, and regional CBV values were calculated by a region of interest analysis between tumor and normal tissue. To observe BBB permeability, vascular constant transfer (*K*^trans^ min^− 1^) values were calculated using syngo.via (Software Product Line for 3D Routine and Advanced Reading Siemens Healthcare). One region of interest was manually positioned on the solid tumoral area with maximal *K*^trans^ value and compared with contralateral white matter.

### Bioinformatic analysis

Processed transcriptomic data form 981 glioma patients (151 astrocytoma, 109 oligoastrocytoma, 159 oligodendroglioma, and 562 glioblastoma) were obtained from the work published by Ceccarelli et al. in 2016 at https://tcga-data.nci.nih.gov/docs/publications/lgggbm_2015/ [28] (Additional file 1: Tables S2-3). The histogram of the VEGFA expression of TCGA (Additional file 1: Figure S8 A) and our cohort (Additional file 1: Figure S8 B) was fitted with two Gaussian functions that represent two populations: high (green) and low (brown) VEGFA expression. The middle point between the positions of the two Gaussian functions was used to define the threshold between both populations.

### Statistics

High-volume data downloaded from the TCGA was managed using the R programming language.

For smaller datasets, statistical analysis was performed using a 2-tailed Student *t* test. Data are presented as means ± standard deviation and were calculated using the software package GraphPad Prism v. 5.0. Statistical values of *P* > 0.05 were not considered significant.

## Results

### VEGFA expression level stratifies human glial tumors

**Fig. 1:**
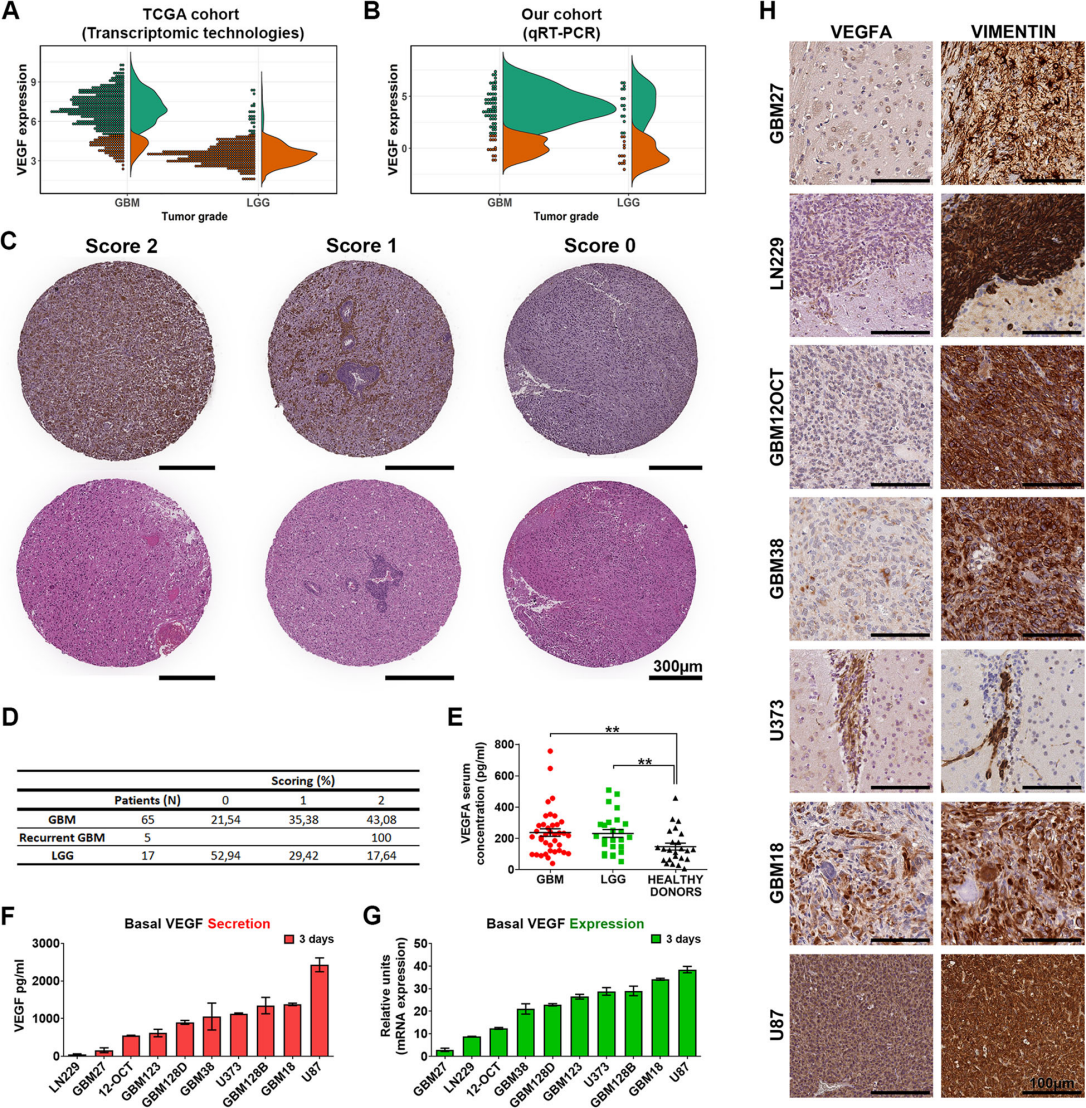
Different levels of VEGFA expression in two human cohorts, in vitro and in xenograft mice. Analysis of VEGFA mRNA expression in gliomas grouped according to histological type and grade **a** by transcriptomic technologies (TCGA cohort, *n* = 981) and **b** by QRT-PCR (our cohort, *n* = 101). Patients were stratified into 2 groups based on VEGFA expression values: high (green) and low (brown). **c** Representative images from the immunohistochemical (IHC) analysis of VEGFA in patient tissue microarrays (TMAs). Samples from epileptic patients were used as controls. Scale bar, 300 μm. **d** Quantification of the IHC analysis of VEGFA expression in our cohort. Staining was scored as follows: 0 = rare, 1 = localized, and 2 = diffuse. Data are expressed as the percentage of the number of positive cases/total number of specimens. **e** VEGFA serum concentration (pg/ml) prior to surgery in GBM and LGG. Healthy donors were used as controls. Student’s *t* test **P < 0.01. **f** Ten glioma cell lines were analyzed for the secretion of VEGFA to the extracellular media at 72 h by ELISA (cells were seeded at 1500 cells/well as a starting culture density). **g** Basal VEGFA mRNA expression from 10 glioma cell lines quantified by QRT-PCR. Data represent the mean ± S.D. of two independent experiments performed in triplicates. **h** Representative IHC images of sections obtained from brain intracranial xenografts using anti-VEGFA and anti-human vimentin. Scale bar, 100 μm

To observe the clinical relevance of VEGFA, we first evaluated its mRNA expression in a cohort of 981 low- (LGG) and high-grade glioma (HGG) patients [28] (Additional file 1: Tables S2-3). Our results show that the level of VEGFA expression increased according to the histopathology degree. Distinct gene expression patterns were seen in the set of GBM samples, in which two different subgroups—high (green) and low (brown) expression—could be distinguished. Notably, there were 20% of GBM patients in the TCGA cohort and 25.9% in our cohort with low VEGFA levels. In the LGG population, VEGFA expression presented predominantly low values (Fig. 1a). Similar data were registered in our GBM cohort (*N* = 73); however, our LGG patients (*N* = 28) showed higher values than TCGA samples (Fig. 1b, Additional file 1: Table S1).

Results on VEGFA IHC staining are shown in Fig. 1c, d. 21.54% cases of de novo GBM and 52.94% of LGG patients showed rare or no expression of VEGFA protein (IHC score 0), 35.38% and 29.42% GBM and LGG respectively were scored as (1) localized and the rest of the patients and all the secondary GBMs had a very high score (2). Although the mean serum VEGFA concentrations (pg/ml) did not differ significantly between GBM and LGG, two secretion clusters could be distinguished in both groups. Moreover, VEGFA secretion values in both glioma groups were higher than in healthy donors (*P* < 0.01) and correlated with the tumor grade (Fig. 1e).

In concordance with the results observed in patients, distinct clusters based on VEGFA secretion and expression were detected in ten GBM cell lines, which showed a high correlation between supernatant VEGFA levels and mRNA values (Fig. 1f, g and Additional file 1: Figure S1). Remarkably, these patterns were reproduced in intracranial xenografts, showing a clear relation between in vitro and in vivo behavior (Fig. 1h). Together, these results demonstrate that glioma patients could be divided according to VEGFA expression and secretion levels.

### Specific but not standard bevacizumab doses impair VEGFA expression and secretion

In view of the importance of VEGFA in the angiogenesis pathway and to better understand its role in GBM, we selected one representative cell line of each subgroup, U87 as high, U373 as medium, and LN229 as low expression.

**Fig. 2:**
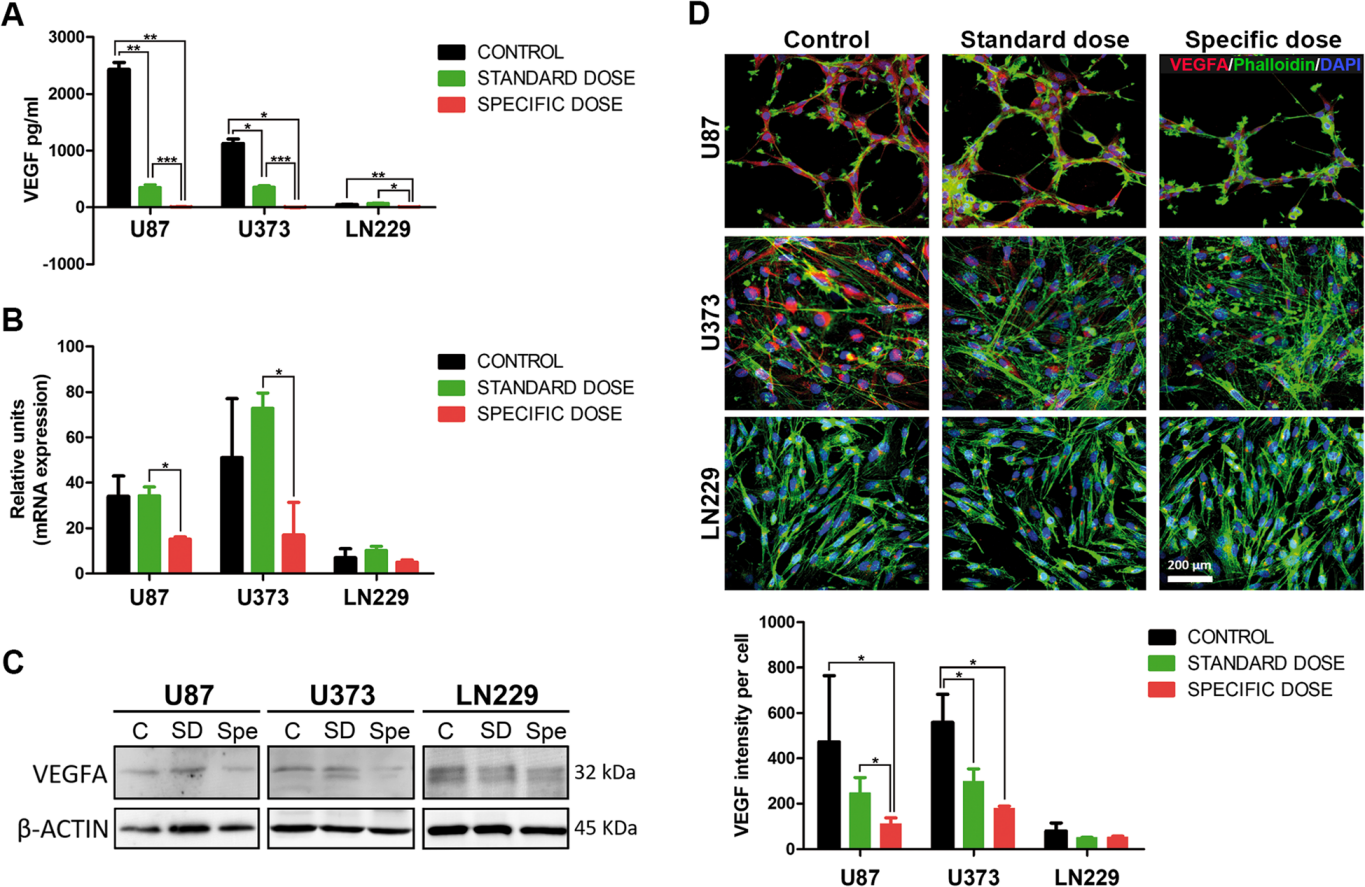
Specific dose of bevacizumab neutralizes VEGFA secretion. **a** VEGFA quantification (pg/ml) at 72 h by ELISA of U87, U373, and LN229 treated with specific dose (22 μg/ml, 10 μg/ml, and 0.42 μg/ml, respectively), standard dose Bev (8.3 μg/ml), and IgG as a control. **b** Evaluation of the relative expression of VEGFA in treated cell lines by QRT-PCR. Ct values were normalized to GAPDH and β-actin. **c** The expression of VEGFA was determined via Western blot after the 72-h treatments. Actin levels were used to normalize. **d** Immunofluorescence staining of actin-phalloidin (green) and VEGFA (red), including merged nuclei (DAPI, blue). Scale bar, 200 μm. VEGFA signal intensity quantification per cell is shown on the bottom. Data are shown as mean ± S.D. and are representative of 2 independent experiments. *P* values were calculated based on the 2-tailed 2-sample *t* test. *P < 0.05, **P < 0.01, ***P < 0.001

The second set of experiments examined the ability of bevacizumab to inhibit VEGFA secretion in vitro at normoxic and hypoxic conditions*.* Although bevacizumab could compete with the detection antibody, ELISA assay is able to measure the total VEGFA present in the extracellular media [29]. For this purpose, we used a standard dose (SD; 8.3 μg/ml) which mimics the clinical administration and a specific dose (Spe) calculated for each cell line (see Additional file 1: methods section). As can be seen in Fig. 2a and Additional file 1: Figure S2, the Spe calculated for each cell line (Additional file 1: Table S4) is able to neutralize VEGFA secretion in the three cell cultures studied. Once we evaluated both conditions, normoxia was established for the following experiments. Although VEGFA presence in the extracellular media was reduced using the SD, its complete inhibition was not reached. Next, we wondered if the inhibition of extracellular VEGFA could have an impact on VEGFA expression levels, as autocrine loop has been described [30]. VEGFA transcript and protein levels were only decreased using Spe in U87 and U373 (Fig. 2b, c). Similarly, SD had only a small effect on cellular VEGFA expression in U87 and U373, but Spe significantly inhibited the VEGFA expressed in these cell lines (Fig. 2d). Bevacizumab Spe did not change the cell morphology (Additional file 1: Figure S3), and no effects were observed in hMSCs viability (Additional file 1: Figure S4) showing low toxicity levels. Our results prove that the neutralization of the secretion of VEGFA is bevacizumab dose-dependent.

### Specific dosage of bevacizumab inhibited HBMEC migration and tube formation in tumor cells

In an effort to elucidate the underlying mechanism of bevacizumab in angiogenesis, we exposed HBMECs to conditioned media (CM) obtained by treating U87, U373, and LN229 cell lines with standard (SD) or specific doses (Spe) of bevacizumab. In each case, IgG were used as a control (C).

**Fig. 3:**
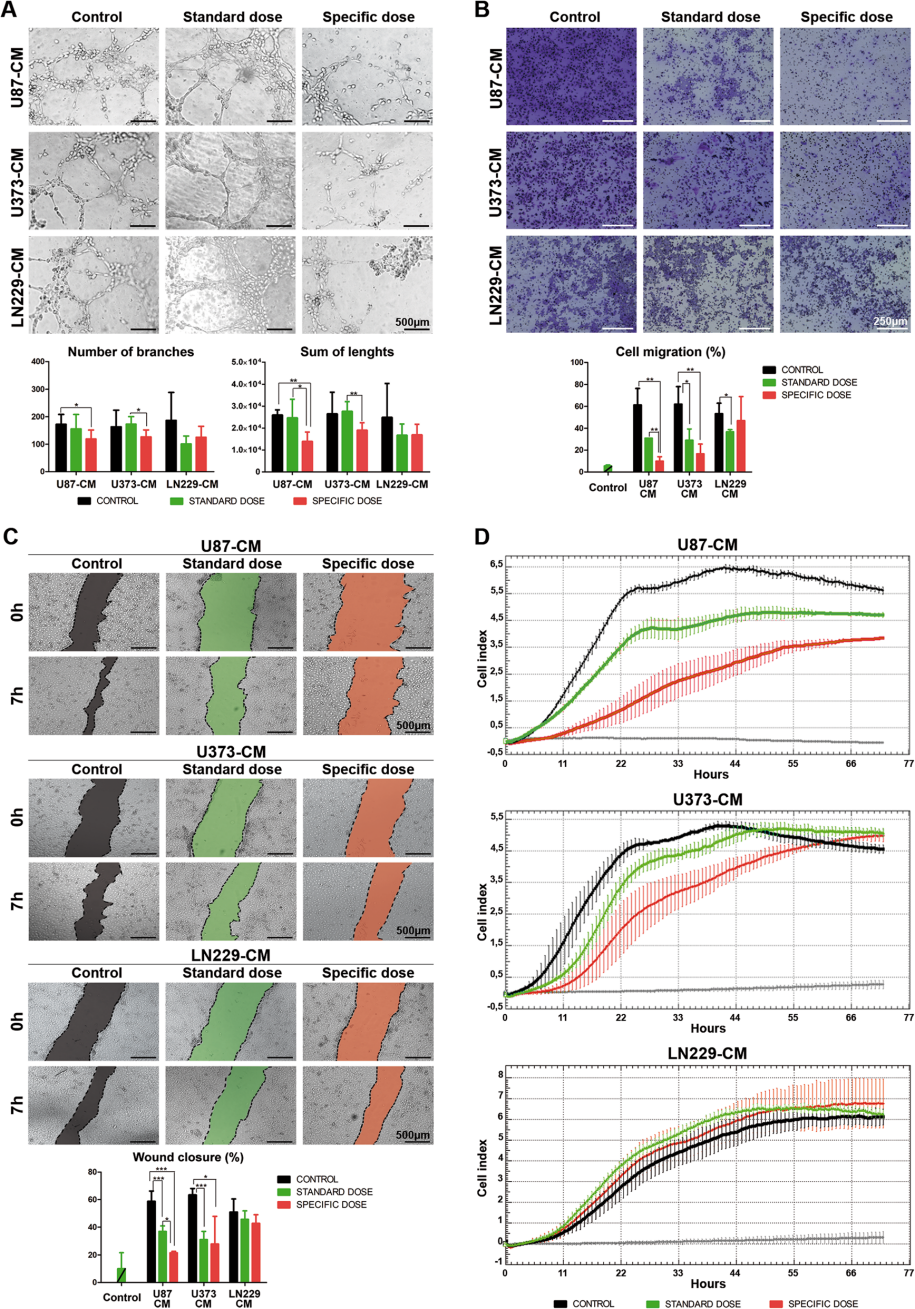
Specific dose of bevacizumab obtained from U87 and U373 inhibited HBMEC angiogenesis in vitro. **a** Representative micrographs show endothelial network formation after 18 h of seeding exposed to conditioned media (CM) from U87, U373, and LN229 treated with IgG (control), standard dose (SD), and specific dose (Spe) of bevacizumab. Scale bar, 500 μm. Quantification (total number of branches and sum of lengths) of HBMEC tube formation from three experiments is shown below. The number of branches only significantly decreased with U87 and U373 Spe-CM (*P < 0.05). The sum of lengths was reduced with U87, U373 SD-CM (*P < 0.05), and Spe-CM (**P < 0.01). **b** Transwell migration assays of HBMEC exposed to conditioned media (CM) and to serum-free media (negative control) (Additional file 1: Figure S5A) (*P < 0.05, **P < 0.01). Scale bar, 250 μm. **c** Wound width was analyzed 0 and 7 h after wounding. Dotted lines indicate the wound borders. Wound closure is expressed as the percentage of the width of the initial wound (HBMEC with serum-free media was used as a negative control, Additional file 1: Figure S5B). Only significant differences were observed in U87 and U373-CM (*P < 0.05 and **P < 0.01, respectively). Scale bar, 500 μm. **d** HBMEC were seeded at 4 × 10^4^ cells per well in uncoated wells. Real-time response curves are data from the xCELLigence Real-Time Cell Analyzer System. The gray curve represents the cells exposed only to serum-free media. Data are shown as mean ± S.D. and are representative of 2 independent experiments, each performed in triplicate. 2-tailed Student’s *t* test

The antivascular potential of bevacizumab was first tested by tube formation assay after 16 h of CM incubation. As shown in Fig. 3a, the branch reduction number was only significant when U87 and U373-Spe-CM were used (*P* < 0.05). Moreover, U87-Spe-CM mediated the inhibition of tube formation compared with the U87-C-CM and SD-CM (*P* < 0.01, *P* < 0.05). In the case of U373-CM, the total network length was reduced using Spe-CM (*P* < 0.01). No effect was observed when LN229-CM was used. These data demonstrate that U87 and U373-Spe-CM can efficiently reduce HBMEC tube formation.

To study the cell migratory behavior, we carried out a transwell migration assay (Fig. 3b). After 3 h, the number of HBMECs that moved through the membrane with U87-C-CM was 6.7-fold higher than the number of cells incubated with U87-Spe-CM (*P* < 0.01). A similar percentage of migrated cells was observed within the U373 group, with statistically significant differences between the U373-C-CM versus SD and Spe (*P* < 0.05).

In the cells incubated with LN229-CM, only a slight reduction was observed when SD-CM was used (*P* < 0.05). These results suggest that the addition of a specific dose of bevacizumab inhibited the migration of endothelial cells.

Furthermore, a wound healing assay showed that the addition of bevacizumab reduced HBMEC motility in the U87 and U373-CM. No changes in cell migration were observed in the wells incubated with LN229-CM (Fig. 3c). Consistent with these observations, we noticed a marked decrease in the HBMEC migration activity using U87 and U373-Spe-CM by the xCELLigence Real Time Cell Analyzer (Fig. 3d) (controls without CM are shown in Additional file 1: Figure S5). Importantly, we found that Spe-CM causes the disruption of the autocrine VEGF/VEGFR2 signaling loop as shown in the dramatic decrease of VEGFR2 expression (Additional file 1: Figure S6).

Altogether, our results indicate a strong decrease in angiogenesis in vitro after the treatment of U87 and U373 cells with a specific dose of bevacizumab.

### Specific dosage inhibits vascularization of Matrigel plugs from in vivo mouse models

**Fig. 4:**
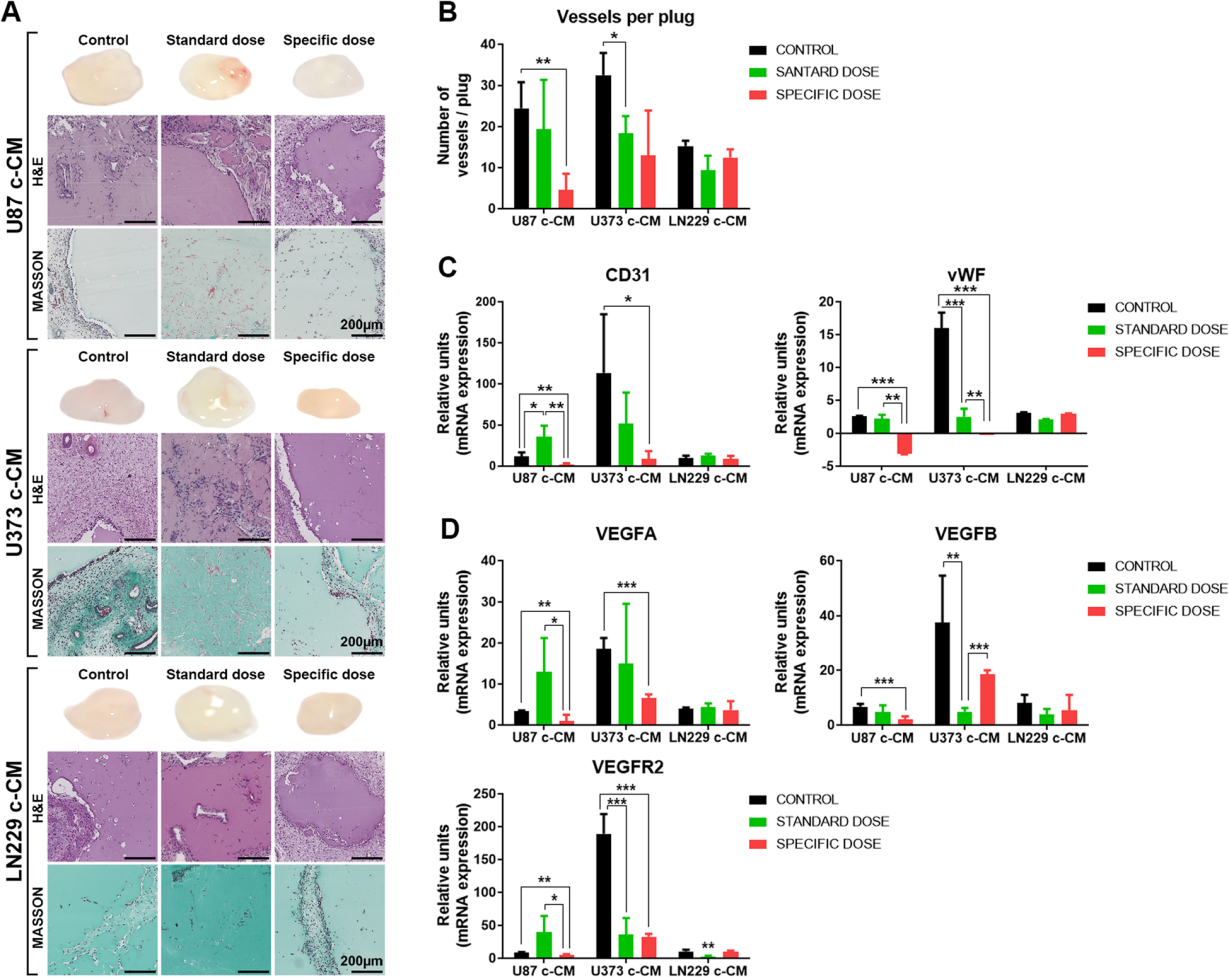
Anti-angiogenic activity of conditioned media using in vivo Matrigel plug assay. Six-week-old nude mice were subcutaneously injected with Matrigel containing concentrated CM (c-CM). **a** After 7 days, mice were sacrificed, and representative Matrigel plugs were removed and photographed. Sections of each Matrigel plug were stained with H&E and Masson’s Trichrome staining. Scale bar, 200 μm. **b**. The number of blood vessels was counted by two independent observers and averaged. Quantitative RT-PCR analysis of **c** CD31 and vWF and **d** VEGFA, VEGFR2, and VEGFB expression levels in Matrigel plugs from implanted mice. Less neovascularization was observed in the Matrigel plug of c-CM-Spe. Data are representative of at least two independent experiments. Data are shown as mean ± S.D. *P < 0.05, **P < 0.01, ***P < 0.001

Once we confirmed that CM from U87 and U373 treated with Spe of bevacizumab is sufficient to switch off the angiogenesis in vitro, we wondered whether the pathway would be limited in vivo*.* We concentrated conditioned media (c-CM) and mixed it with Matrigel and heparin. Then, it was injected into the mouse flanks. Macroscopic control plug images appeared red, whereas plugs treated with SD and Spe appeared pale in the three cell lines studied. The components of the cellular infiltrate could be observed in H&E and Masson images (Fig. 4a). The histological analysis demonstrated that the Spe markedly reduced blood vessel growth compared with the control group in the c-CM obtained from U87 and U373. Furthermore, the c-CM from U87 Spe and U373 SD and Spe, but not from LN229, presented a sustained decrease in neovascularization (Fig. 4b), which was confirmed in the significant reduction of specific markers for mouse endothelial cells such as CD31 and von Willebrand factor (VWF) (Fig. 4c). SD had no inhibition effect in U87. On the contrary, we observed an increase in endothelial marker expression (Fig. 4c, d). Analysis of angiogenesis-related factors (VEGFA, VEGFB, and VEGFR2) in Matrigel plugs obtained from c-CM-U87 and c-CM-U373 evidenced a high decrease in expression after the addition of the Spe of bevacizumab. We observed that only the addition of the Spe reduced VEGFA expression, while the use of both bevacizumab concentrations diminished VEGFB and VEGFR2 expression (Fig. 4d). These data confirm that the Spe of bevacizumab is needed to inhibit angiogenesis in vivo.

### Specific dosages showed antitumor activity in angiogenic cell lines

**Fig. 5:**
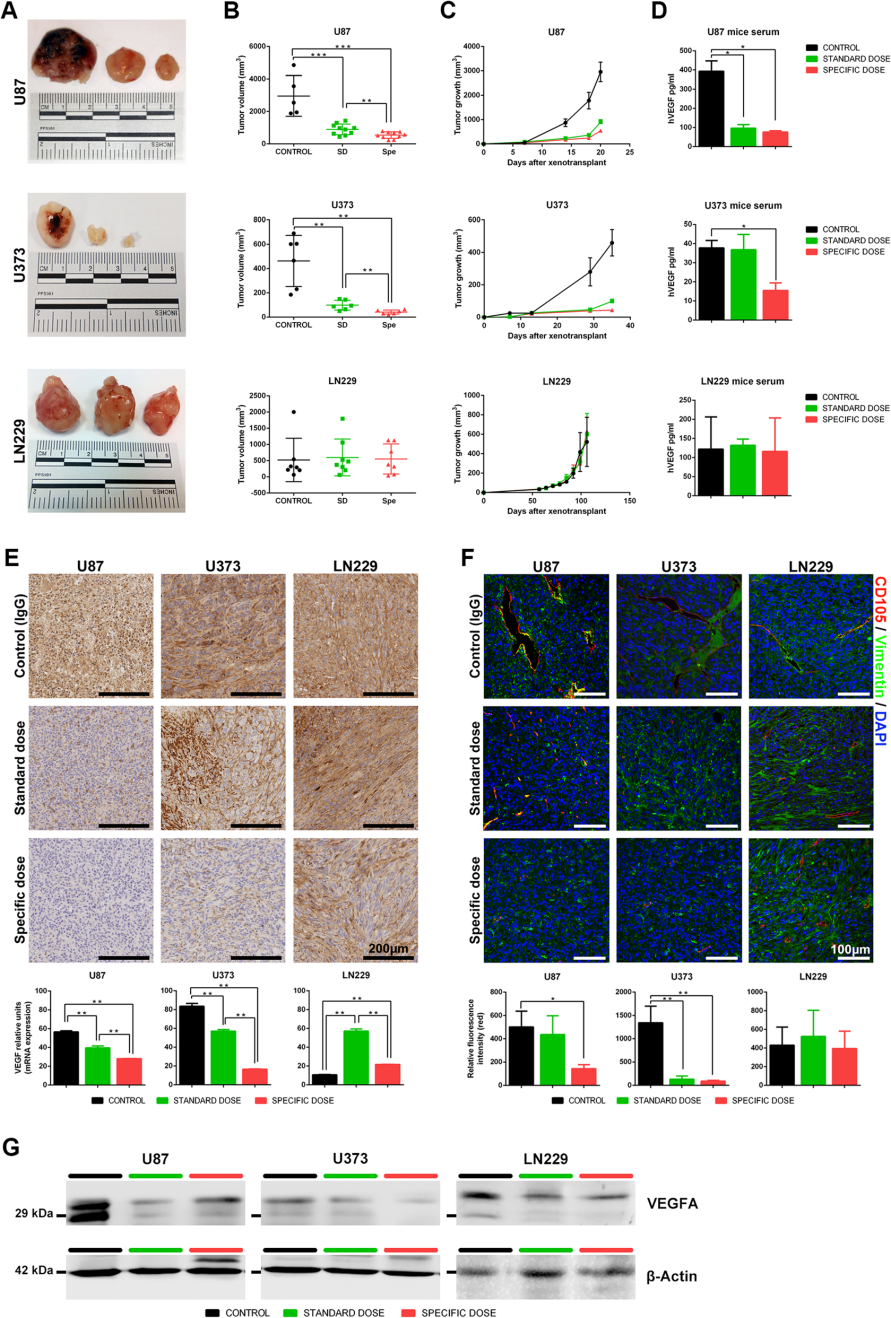
In vivo tumor growth inhibition by a specific dose of bevacizumab in high VEGFA secreted cell lines. **a** 1.5 × 10^6^ U87, U373, and LN229 cells were implanted into the flanks of nude mice. Mice were treated with IgG (*n* = 10), SD (*n* = 5), and a specific dose of bevacizumab (*n* = 5) twice a week. Representative macroscopic tumor images of control (left), SD, and Spe (right) are shown. **b** Tumor volume (mm^3^) at the endpoint. **c** Tumor growth curves. **d** Human VEGFA measure (pg/ml) from mouse serum. **e** Immunohistochemistry images using anti-VEGFA antibody, scale bar 200 μm, and real-time PCR analysis assessing VEGFA expression in tumoral tissue. **f** Immunofluorescence microscopy using anti-human vimentin (green) and mouse CD105 (red). The nuclei were stained with DAPI (blue). Scale bar, 100 μm. Relative fluorescence intensity was calculated for CD105. **g** Western blots demonstrating that Spe of bevacizumab inhibits VEGFA in U87 and U373 cell lines. Results are shown as mean ± S.D. *P < 0.05, **P < 0.01, ***P < 0.001

To further confirm that the Spe of bevacizumab could only reduce tumor growth in high angiogenic cell lines, we implanted U87, U373, and LN229 subcutaneously into the flanks of nude mice. Bevacizumab treatment was administered twice a week following Baumgarten’s recommendations for dose extrapolations between human/iv and mice/ip [27]. Remarkably, U87 and U373 tumor volume and growth were drastically suppressed by SD and in a more significant manner with Spe. However, bevacizumab treatment did not affect tumoral activity in the in vivo xenograft models formed by the low VEGFA-expressed cell line LN229 (Fig. 5a–c). More importantly, the amount of VEGFA secreted by only tumoral cells was quantified from mouse serum using a human-specific test. Values obtained demonstrate that SD could only reduce the amount of VEGFA ligand in the U87 xenograft model, whereas the Spe extended its capability to U87 and U373 models (*P* < 0.05). No effect was detected in LN229 tumors (Fig. 5d).

VEGFA protein expression in tissue was reduced in a dose-dependent manner in U87 and U373 (*P* < 0.01). Surprisingly, bevacizumab treatment increased VEGFA expression in LN229 tissue sections (Fig. 5e). The study of new blood vessel formation by CD105 confirms that bevacizumab treatment is only effective in cell lines with high VEGFA secretion (Fig. 5f), confirmed by the substantially reduced protein levels compared to the control (Fig. 5g). H&E staining revealed no changes in cell morphology; however, U373-xenografted mice treated with Spe had lower cellularity (Additional file 1: Figure S7). Taken together, our results suggest that two different groups of patients could be stratified depending on basal VEGFA secretion.

### VEGFA serum concentration key to predict the response to bevacizumab therapy in glioma patients

**Fig. 6:**
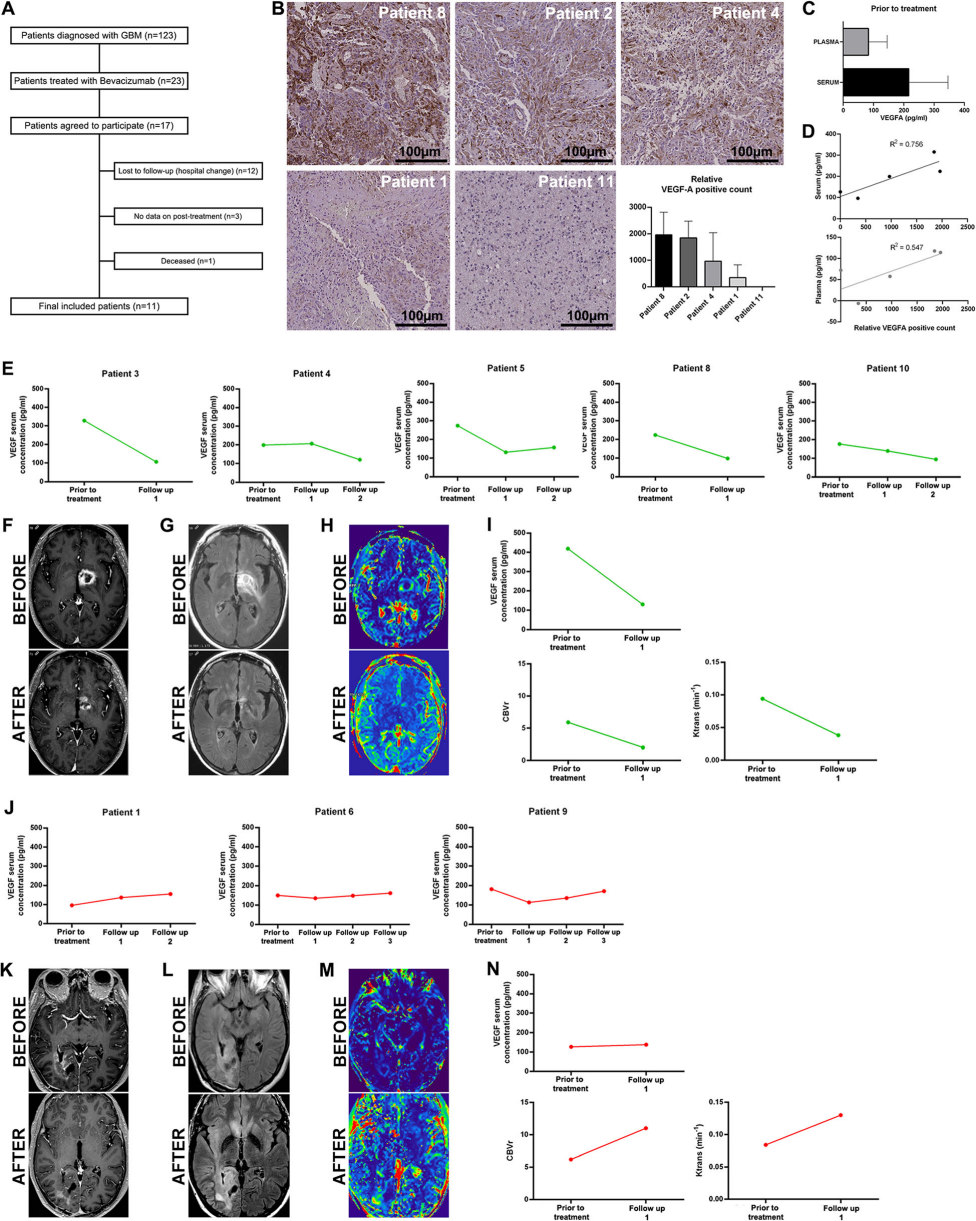
VEGFA serum values correlate with tissue expression and radiographic response. **a** Pre-clinical trial design scheme. **b** IHC representative images using anti-VEGFA antibody in brain tumor patients (scale bar, 200 μm). Relative VEGFA quantification from tumor sections. Ten fields per section were used to quantify VEGFA-positive counts. Bars represent the relative mean values ± S.D. **c** Basal VEGFA levels in the plasma and serum obtained from GBM patients prior to bevacizumab treatment (pg/ml). **d** Correlation between VEGFA values in surgical biopsy and serum (*R*^2^ = 0.756, *P* < 0.05) and plasma (*R*^2^ = 0.547, *P* = 0.153). **e** VEGFA serum values prior to bevacizumab treatment and follow-up (pg/ml) in responder patients. **f**–**i** Axial postcontrast 3D T1-weighted images (**f**), FLAIR T2-weighted images (**g**), and DCE (**h**) perfusion parametric maps of patient 7 prior (upper) and after (bottom) treatment with bevacizumab illustrate tumor response. **i** A decrease in serum values and on rCBV and *K*^trans^ value. **j** VEGFA serum values prior to bevacizumab treatment and follow-up (pg/ml) in non-responder patients. **k**–**n** Axial postcontrast 3D T1-weighted images (**k**), FLAIR T2-weighted images (**l**), and DCE (**m**) perfusion parametric maps of patient 11 prior (upper) and after (bottom) treatment with bevacizumab demonstrate tumor progression. **n** An increase in serum values, on rCBV and *K*^trans^ value

In order to accomplish our study objective, we selected the patients treated with bevacizumab following the scheme depicted in Fig. 6a. The results obtained from surgical solid biopsy support the idea that some GBM patients have a tumor with a high angiogenic phenotype (patients 8 and 2), while in others, VEGFA protein expression is absent (patient 11) (Fig. 6b). Considering that there is no consensus for VEGFA measurement in the peripheral blood, we evaluated it in plasma and serum, and we observed that the serum mean concentration was almost 2.5 times the plasma value (Fig. 6c). Interestingly, the correlation between VEGFA expression in tumor tissue and serum (*r* = 0.87, *P* < 0.05) was much stronger than between the levels in tumor tissue and plasma (*r* = 0.75, *P* = 0.153) (Fig. 6d). To further confirm this observation, we collected blood prior to and after bevacizumab treatment. As expected, a strong association was shown between VEGFA serum levels, radiographic response, and RANO criteria (Additional file 1: Table S5). These values correlate with CBVr and *K*^trans^ and could distinguish between potential bevacizumab responder (Fig. 6e–i) and non-responder patients (Fig. 6j–n and Additional file 1: Figure S9). No association was observed in the plasma (Additional file 1: Figure 1S0).

## Discussion

Glioma growth and progression are dependent on neovascularization [31], which involves endothelial cell proliferation, migration, and tube formation [32]. Since VEGFA is the main pro-angiogenic factor, it has been used as a target in several pathologies [33]. However, the clinical benefit in glioma patients has been questioned considering the results obtained in clinical trials [34], raising much controversy [35]. Some possible explanations for the lack of therapeutic effects of bevacizumab are the acquired resistance observed in some patients and the fact that more than 30% of placebo arm patients received bevacizumab during disease progression [36]. From our in silico analysis of TCGA data, we observed two different populations based on VEGFA expression which were confirmed in mRNA, protein, and serum levels in our own cohorts. The data suggest that there are more than 20% of GBM patients whose tumor growth is independent of the VEGFA pathway, so they would not benefit from an anti-angiogenic treatment.

Our expression results support the idea that GBM tumors are more angiogenic than LGG [37] and even more remarkably in recurrent GBM. These differences were not detected in the peripheral blood, where we observed similar VEGFA levels between HGG and LGG patients. However, significant differences were observed compared to healthy donors, confirming that brain tumor patients release more VEGFA into the bloodstream [38].

As bevacizumab is an antibody that neutralizes the cytokine VEGFA, we wondered if the benefit from this agent could correlate with its tumor expression pattern. Our results showed that there is a group of cell lines with low VEGFA expression/secretion (GBM27, LN229, 12OCT) that would not be candidates for this anti-angiogenic treatment as no response was observed with standard and specific bevacizumab doses in the cell line that represents that group (LN229). Moreover, VEGFA expression profiles from these cell lines are maintained in in vivo models, pointing to their potential use as pre-clinical models [39]. Focusing on the cell lines that would respond to bevacizumab therapy, VEGFA transcript levels were only significantly reduced using a specific dose, which highlights the need to optimize antibody dosing [40, 41]. In this sense, a recent study has highlighted that adjusting doses of bevacizumab could increase concomitant chemotherapy delivery [42].

It is known that VEGFA directs binding to its receptor VEGFR2 and stimulates angiogenesis, endothelial cell migration, and cancer cell proliferation and survival [43, 44]. Our study confirms that in U87 and U373 GBM cells treated with the standard dose of bevacizumab, which mimics clinical administration, the migration of brain endothelial cells decreases in vitro and in vivo, with no effect observed in VEGF/VEGFR2 signaling. The inhibition of this switching loop together with the significant reduction in endothelial marker expression and HBMEC migration was only reached when high VEGFA cancer cell lines were treated with the specific dose. As no effect was detected in the LN229 cell line, we speculate that cell lines with a low VEGFA expression/secretion values could correspond with non-responder patients. These data suggest that VEGFA values could be used as a predictor of bevacizumab dose, increasing its therapeutic efficacy.

In this context, one of the challenges is to distinguish between VEGFA released from tumoral cells and other sources [45]. The amount of hVEGFA measured in the serum from our xenograft mouse models corroborates that a specific dose was more effective than the standard dose in pro-angiogenic cell lines. We show that this treatment impairs tumor growth and results in less vasculature. In contrast, none of the bevacizumab dosages had an effect on the cell line with low VEGFA values, confirming our in vitro results. These data have several therapeutic implications, as some patients might not require bevacizumab treatment, while others need an adjusted dosage.

In this regard, several approaches have been studied to identify which patients would benefit from this treatment, for example, VEGFA plasma levels were measured prior to treatment in AVAGLIO cohort [46], and high VEGFA expression in tissue was correlated with the radiographic response in some patients [47]. However, no association was observed with patient outcomes. In contrast, we and others have shown that VEGFA plasma values do not represent the real angiogenic tumor status [48]. For this reason, as there are no established biomarkers to predict response to bevacizumab therapy, we propose the analysis of VEGFA in the original tissue and serum to help in patient stratification and to identify the mechanisms of resistance and escape routes [49, 50]. Moreover, the analysis of VEGFA in serum could help monitor disease progression and treatment efficacy, supporting MRI data.

Future experiments monitoring VEGFA serum levels with larger cohorts would be required to confirm these effects and to observe differences in the OS, and although our small cohort is highly representative, bevacizumab is not approved by the European Medicines Agency (EMA) [51]. To overcome this issue, we analyzed the OS in the GBM-IDHwt TCGA cohort and divided the cohort based on the VEGFA expression into high and low clusters (Additional file 1: Table S6). Interestingly, when we divided the cohort according to treatment criteria, we did not see differences in OS in the full population (Additional file 1: Figure S11A). However, the median OS of patients with high VEGFA treated with bevacizumab increased in 8.5 months (Additional file 1: Figure S11C), and no effect was observed in patients with low VEGFA expression (Additional file 1: Figure S11B).

Although this is only a proof of concept, since those patients received a multimodal therapy including several drugs, this novel approach could indicate a change in the paradigm of GBM patients’ care.

## Conclusion

Our results show that adjusting bevacizumab posology could be a successful strategy to further predict response stratification. Moreover, we propose to measure the amount of VEGFA in serum and not in plasma to enhance treatment efficacy.

## Supplementary information

**Additional file 1:.** Supplementary tables, figures, and methods.

## Data Availability

Not applicable.
