# Supplementary material for: Bevacizumab dose adjustment to improve clinical outcomes of glioblastoma
Source: BMC Med. 2020 Jun 22;18:142. doi: 10.1186/s12916-020-01610-0 (PMC7310142; doi:10.1186/s12916-020-01610-0)
Supplement: Supplementary file 1 — Additional file 1:. Supplementary tables, figures, and methods. [file 12916_2020_1610_MOESM1_ESM.docx]

**TITLE: Bevacizumab Dose Adjustment to improve clinical outcomes of glioblastoma**

**AUTHORS:** García-Romero N^1^, Palacín-Aliana I^1^, Madurga R^1^, Carrión-Navarro J^1^, Esteban-Rubio S^1,2^, Jiménez B^1^, Collazo A^1^, Pérez-Rodríguez F ^1^, Ortiz de Mendivil A^1^, Fernández-Carballal C ^3^, García-Duque S^1^, Diamantopoulos-Fernández J^1^, Belda-Iniesta C ^1.^ Prat-Acín R ^4^, Sánchez-Gómez P ^5^, Calvo E ^1,6^, Ayuso-Sacido A ^1, 2*^.

**Additional file: methods section**

**Bevacizumab calculations**

Bevacizumab specific doses were calculated as follows: different doses of bevacizumab (1 ng/ml, 10 ng/ml, 100 ng/ml, 1 µg/ml, 10 µg/ml, 0.1 µg/ml, 0.25 µg/ml) were used in U373 cell line to inhibit VEGFA ligand at 72 h. Data were extrapolated to other cell lines, and confirmed *in vitro.* Its inhibition was observed by ELISA (pg/ml). Standard dose (8.3 µg/ml) was calculated according to Baugmarten *et al.,* 2011 in mice (16,6 mg/kg) and then extrapolated into *in vitro* values.

**Morphology assay**

U87, U373 and LN229 were treated for 72 hours with Spe Bev and IgG. Cells were fixed for 5 min in 4% paraformaldehyde, permeabilized for 20 min with 100% methanol and then, stained with hematoxylin.

**Viability assay**

hMSCs (3000 cells/well) were plated in a 96 well plate in a final volume of 100 µl /well. Different bevacizumab concentrations (SD, U87 Spe, U373 Spe, LN229 Spe) were analyzed. 20 µl /well of MTS (CellTiter 96 AQueous One Solution Cell Proliferation Assay, Promega) were added to the culture media, incubated at 37ºC for 2 hours and absorbance was measured at 490 nm. IgG was used as a control. Each treatment group was repeated in triplicate and each experiment in duplicate.

**Additional file: Table S1.** AAS cohort. Tissue and serum samples used in the present work.

**
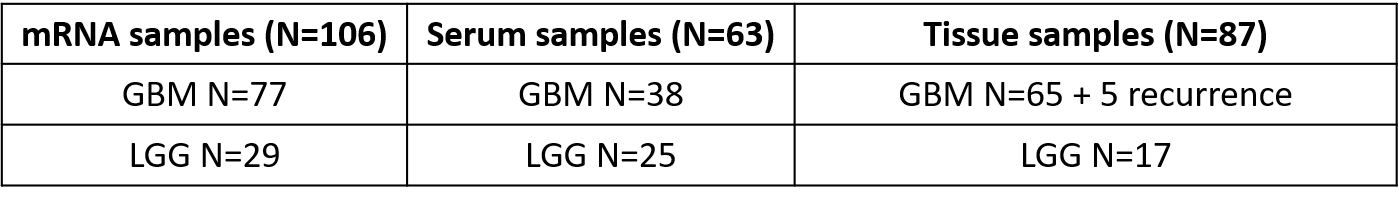
**

** Additional file: Table S3.** TCGA cohort (N=981) obtained from the work published by Ceccarelli *et al.,* in 2016 used in transcriptomic analysis.

**Additional file: Table S4.** Bevacizumab specific doses used in normoxic and hypoxic conditions.

| **Cell line** | **Specific dose Normoxia** | **Specific dose Hypoxia** |
| --- | --- | --- |
| **U87** | 22 µg/ml | 30 µg/ml |
| **U373** | 10 µg/ml | 17 µg/ml |
| **LN229** | 0.420 µg/ml | 5 µg/ml |

**Additional file: Table S5.** Patient characteristics and response assessment.

**
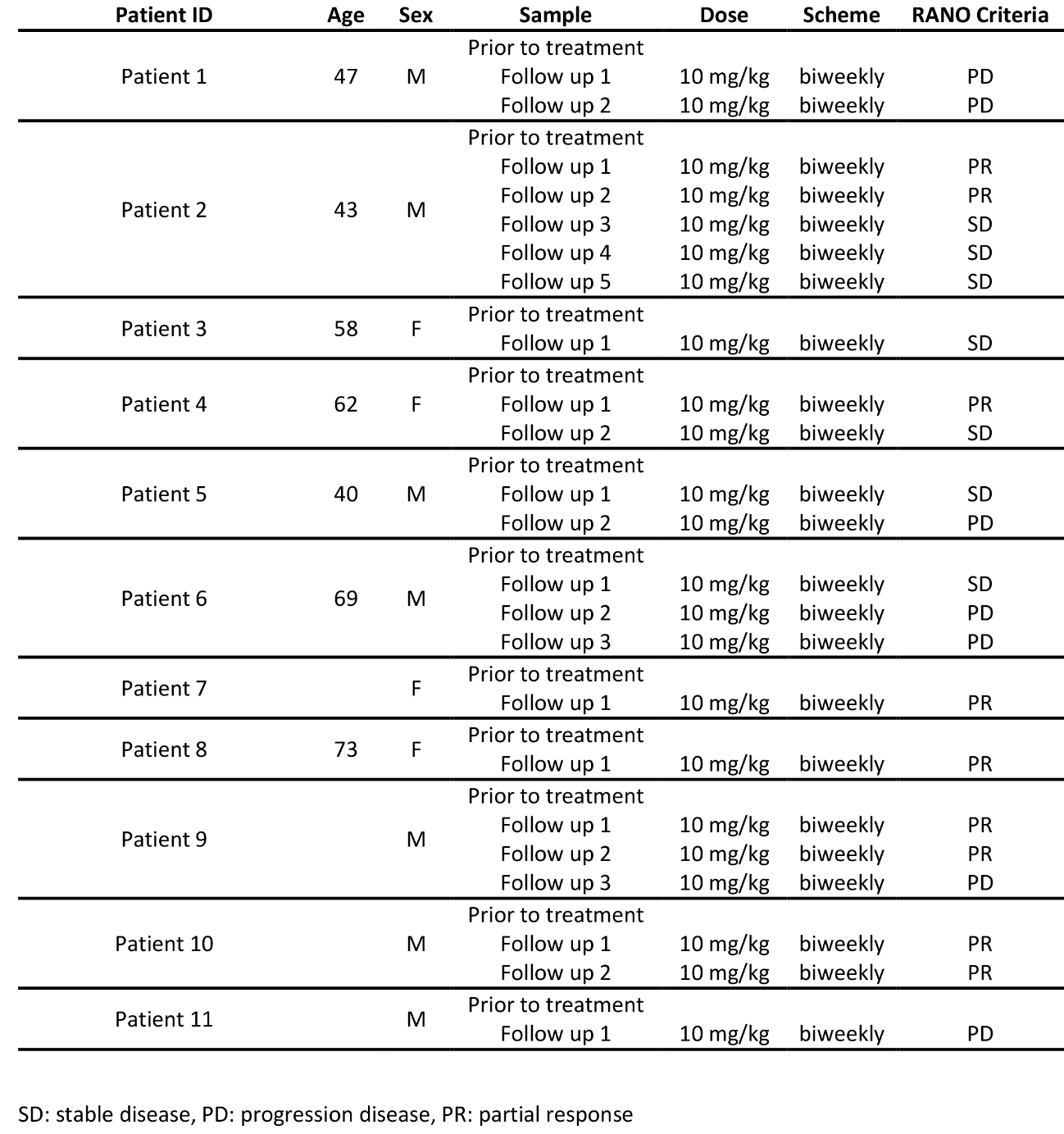
**

**Additional file: Figure S1.** Linear regression observed between VEGFA mRNA expression and secretion values (pg/ml) (R=0.8875) *in vitro*. Ten GBMcell lines were used.

**
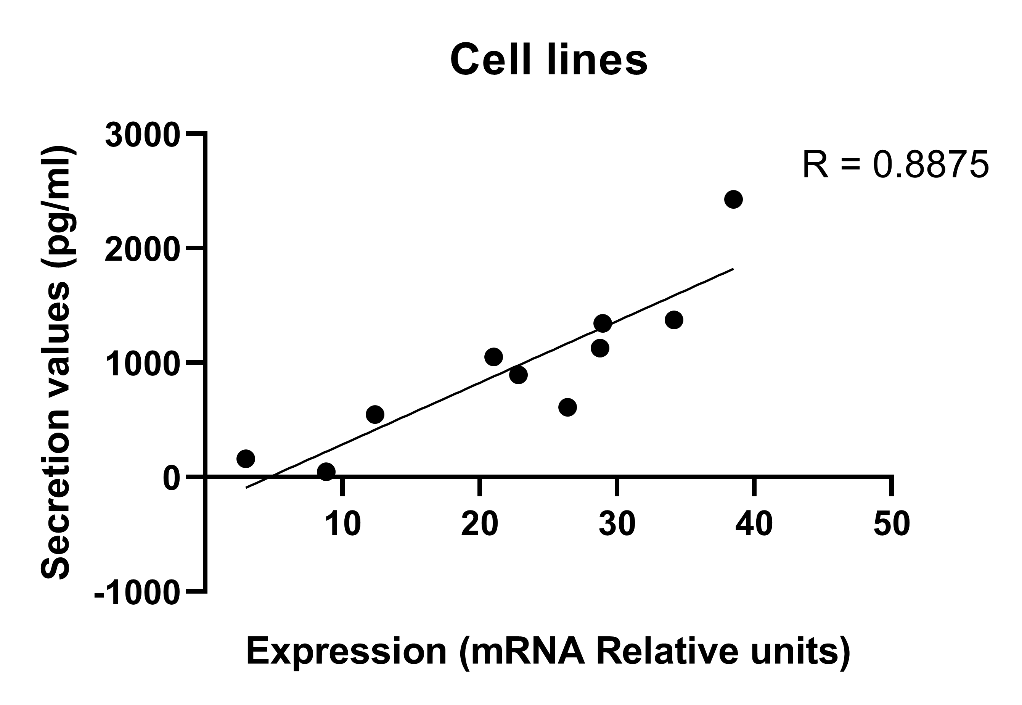
**

**Additional file: Figure S2.** VEGFA quantification (pg/ml) at 72h by ELISA of U87, U373 and LN229 treated with specific dose (30 µg/ml, 17 µg/ml and 5 µg/ml respectively) in hypoxic conditions (1% O_2_). Same amount of IgG was used as control.

**
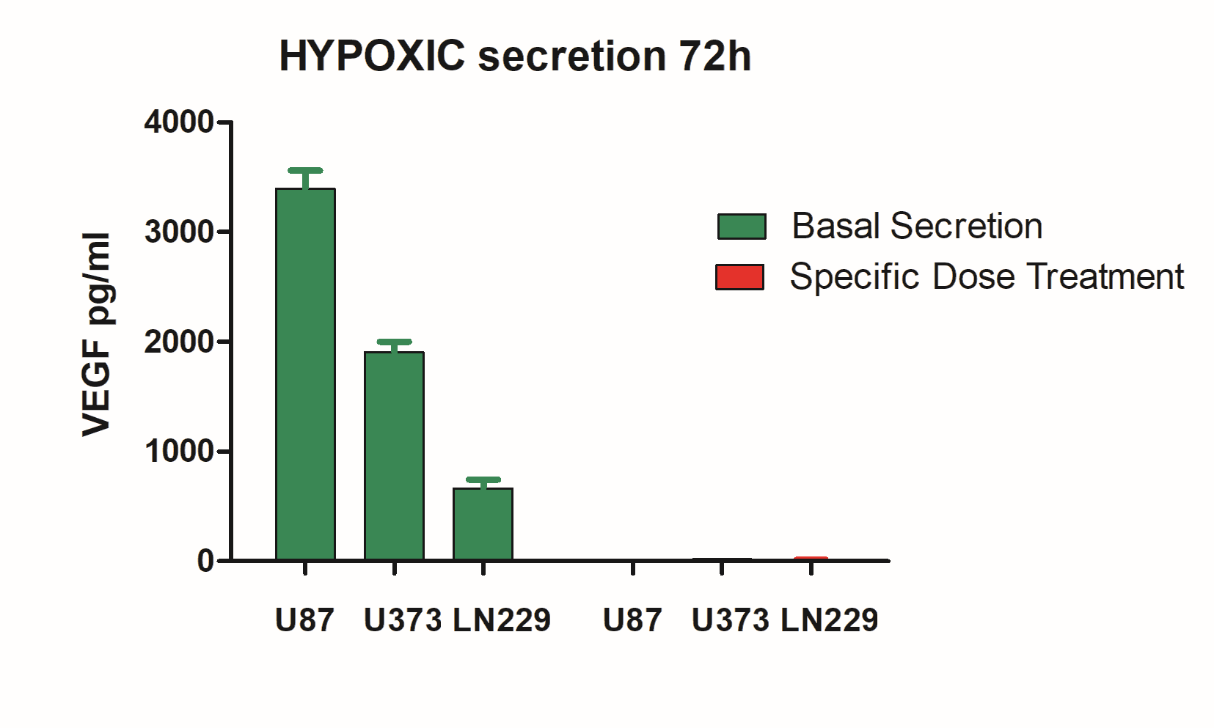
**

**Additional file: Figure S3.** Cell morphology was observed by Hematoxylin at 72h.

**
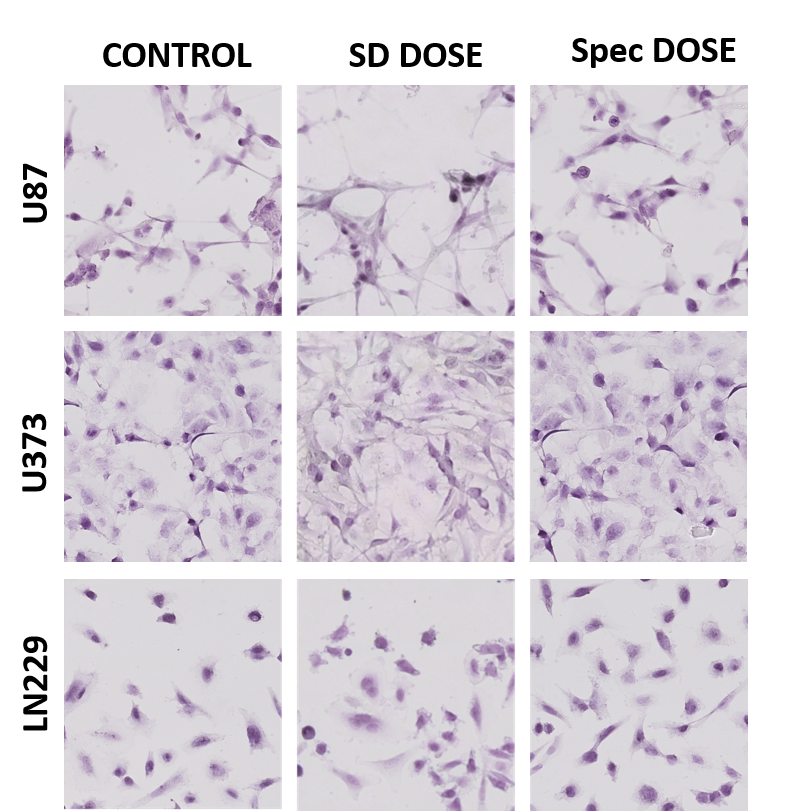
**

**Additional file: Figure S4.** Viability assay for mesenchyal cells treated with SD, U87 Spe, U373 Spe and LN229 Spe dose. Assays were performed in triplicate and repeated twice. Mean ± SD.

**

**

**Additional file: Figure S5.** (A) Control used in transwell migration assay. HMBEC cells were seed with DMEM. (B) Control used in wound healing assay. HMBEC cells were seed with DMEM.

**
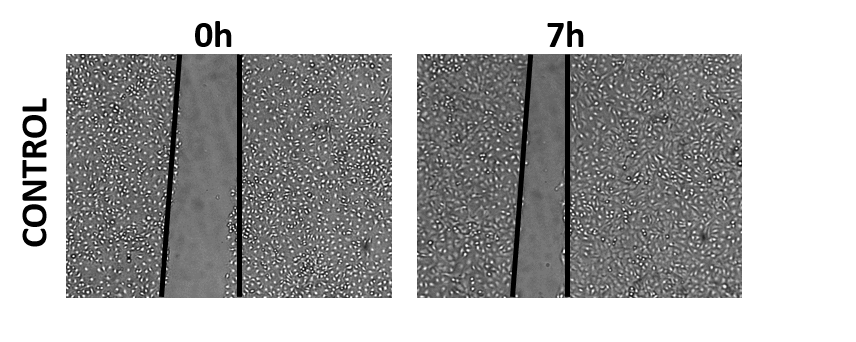

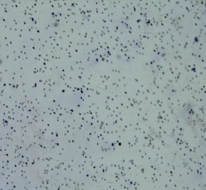
A B**

**Additional file: Figure S6.** VEGFA/VEGFR2 relative mRNA expression observed in HMBEC treated with Conditioned Media. Mean ± SD. P values were calculated based on the 2-tailed 2-sample t test. * P<0.05, **P<0.01, ***P<0.001.

**
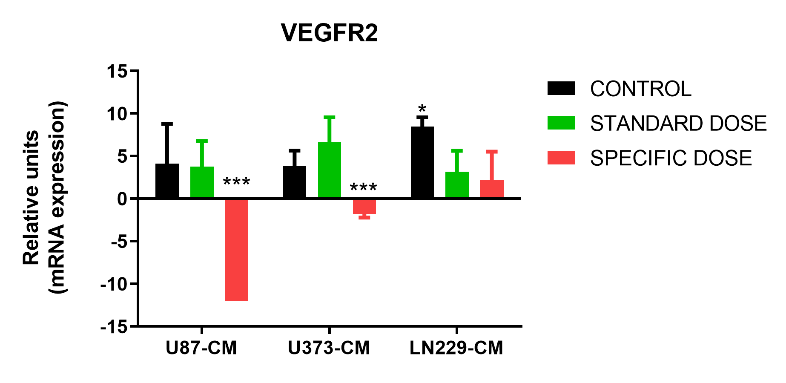

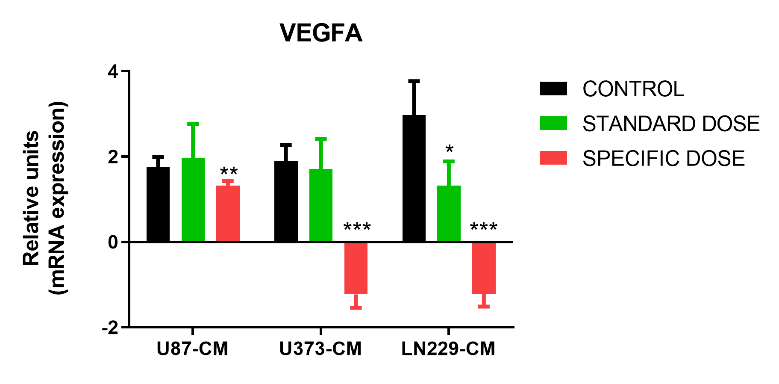
**

**.**

**Additional file: Figure S7.** H&E stained sections from xenotransplanted mice treated with and without bevacizumab. Scale bar =100 µm.

**
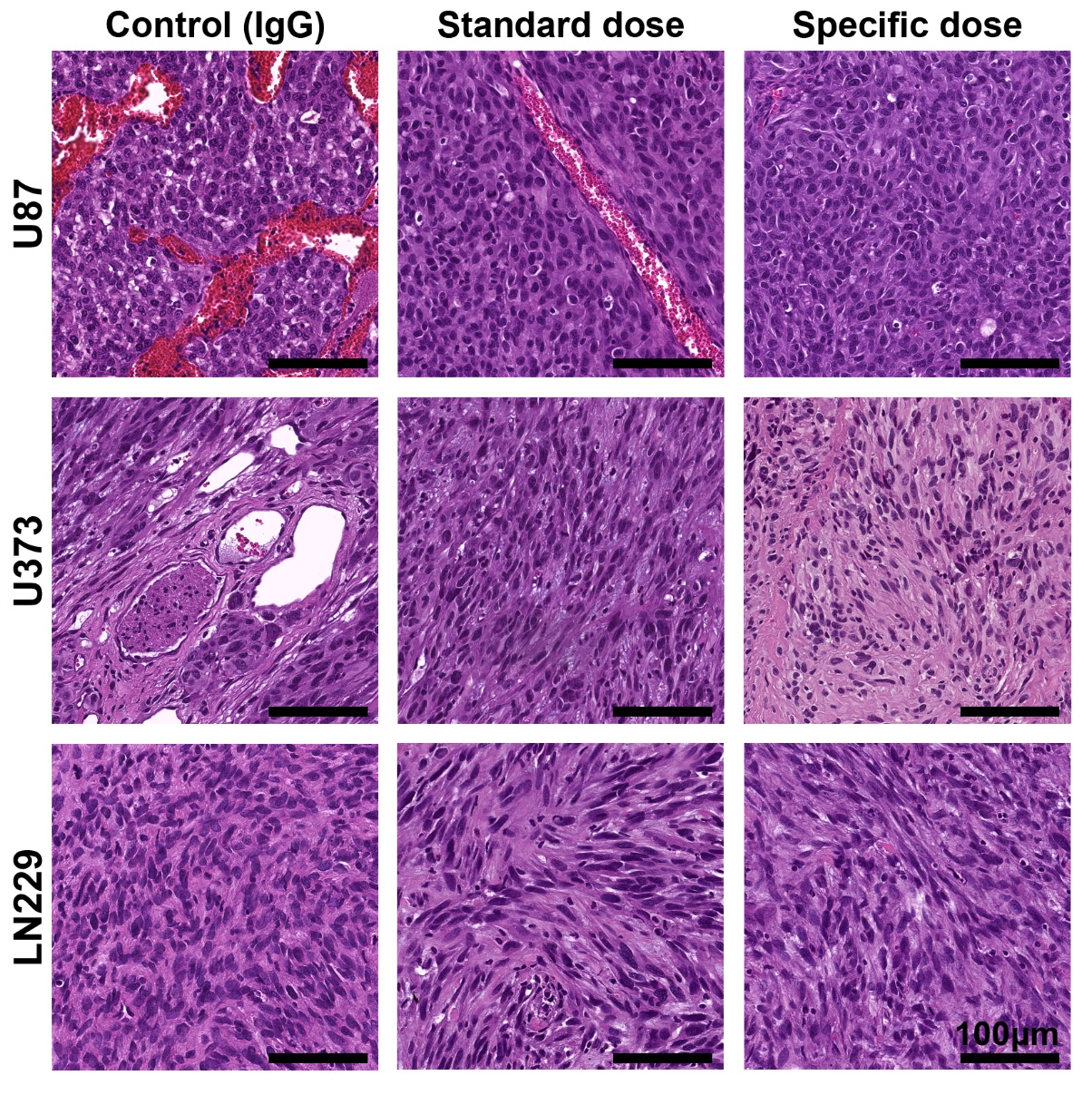
**

**
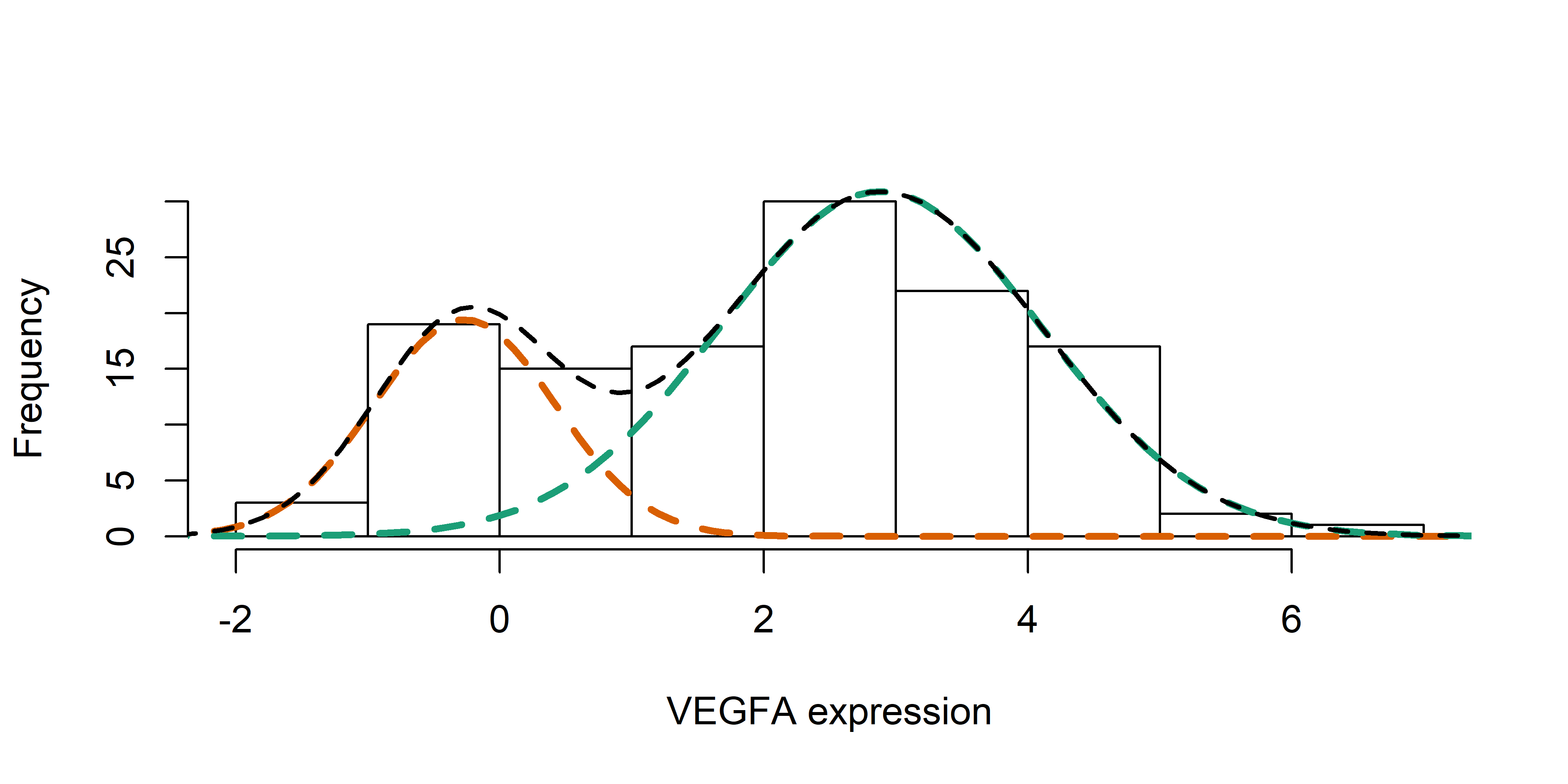
 Additional file: Figure S8.** Histograms of the VEGFA expression in TCGA **(A)** and our cohorts (AAS) **(B)** were fitted with two Gaussian functions that represent two populations: high (green) and low (brown) VEGFA expression. The middle point between the position of the two Gaussian functions was used to define the threshold between both populations.

**A**

**
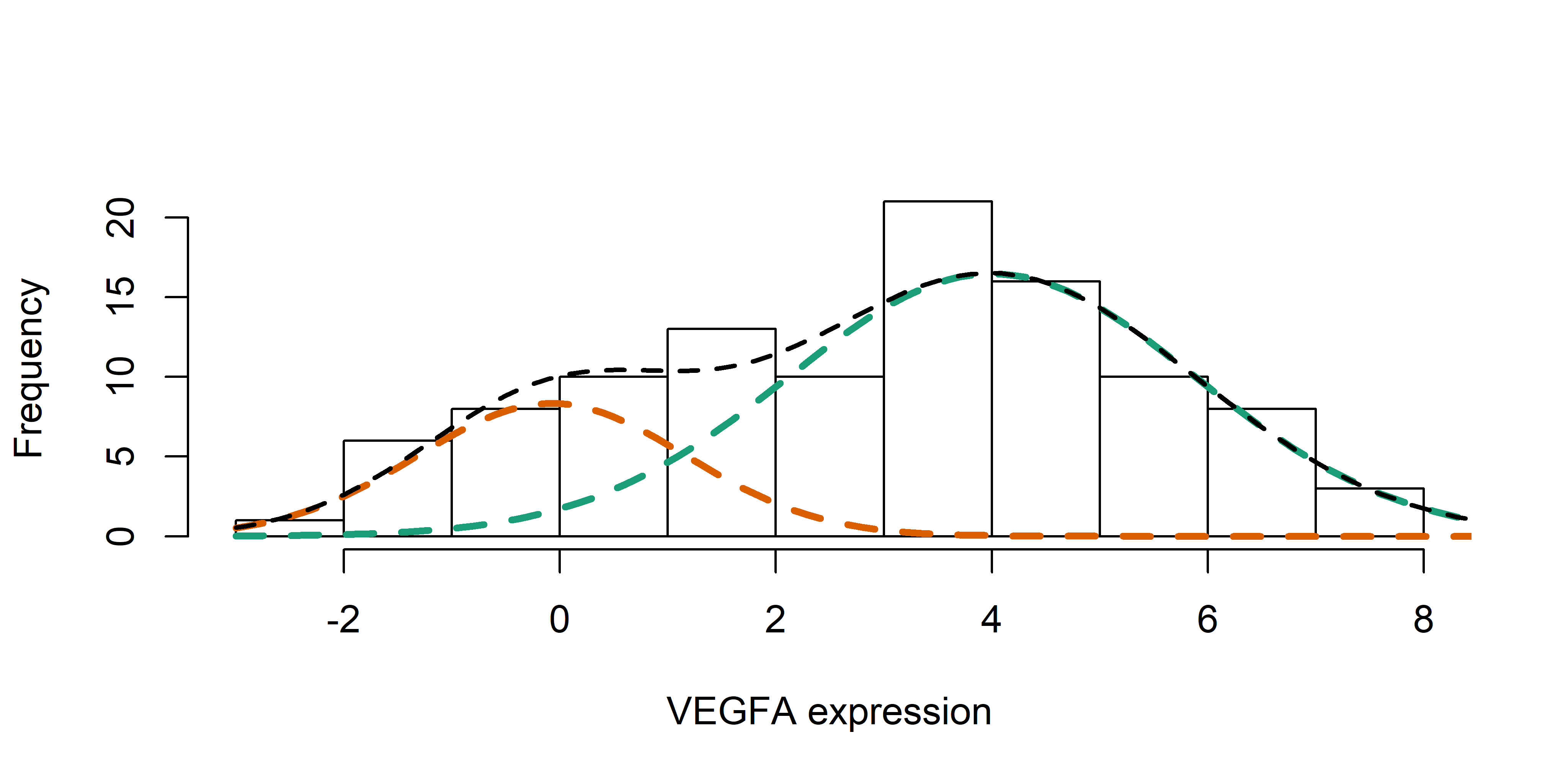
B**

**Additional file: Figure S9.** Axial DSC and DCE perfusion parametric maps and morphologic images on patient 2 prior to surgery (upper) and after bevacizumab treatment (bottom). Pretreatment (upper) and postreatment (bottom) CBV map and postcontrast T1-weighted image showing ROI analysis comparing maximal tumoral CBV compared with contralateral tissue. Dynamic Ktrans map and postcontrast T1-weighted images depicting ROI analysis (maximal tumoral/contralateral tissue).

**
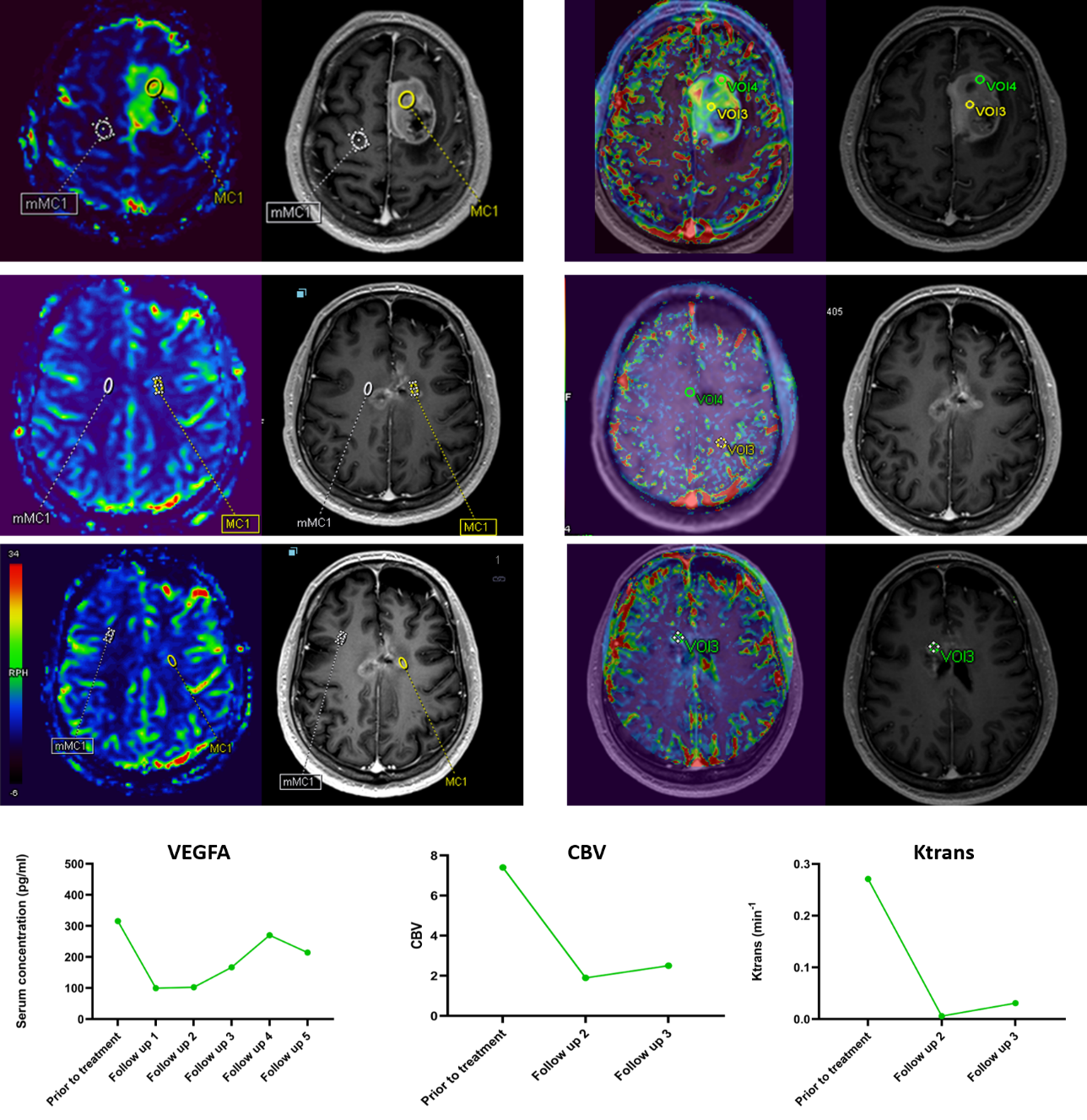
**

**Additional file: Figure S10.** VEGFA plasma values in patients prior to bevacizumab treatment and follow-up (pg/ml).

**
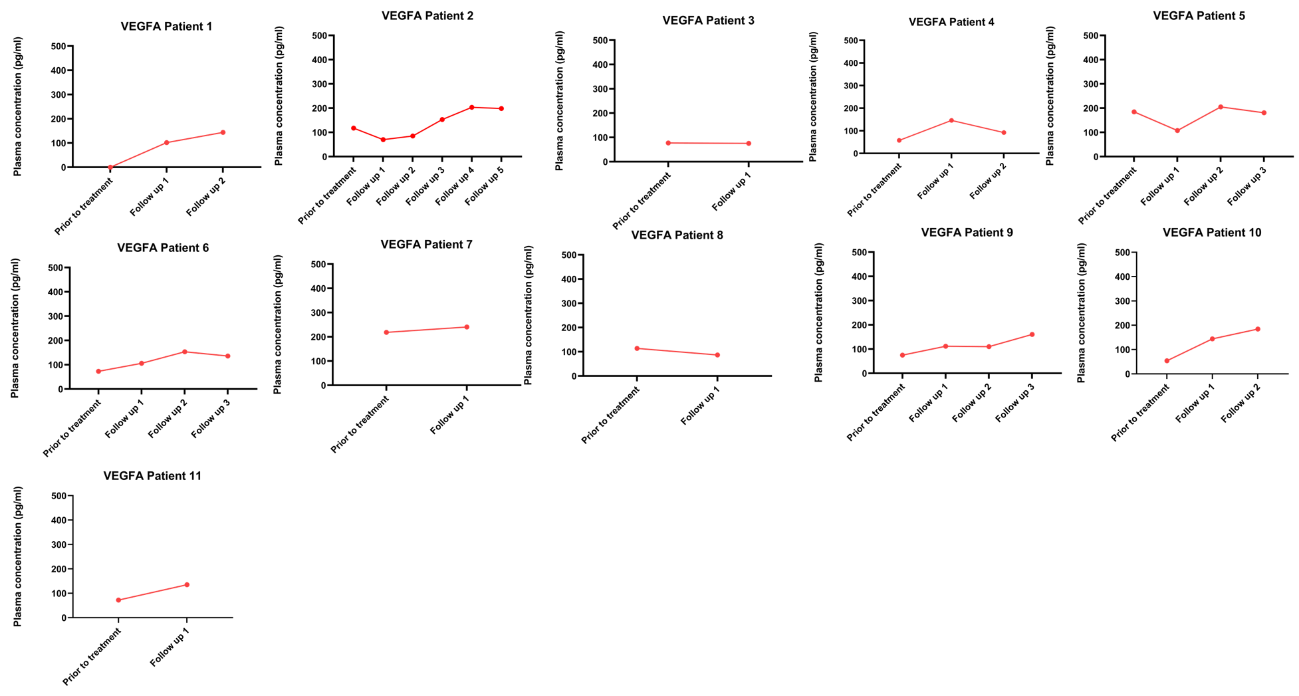
**

**
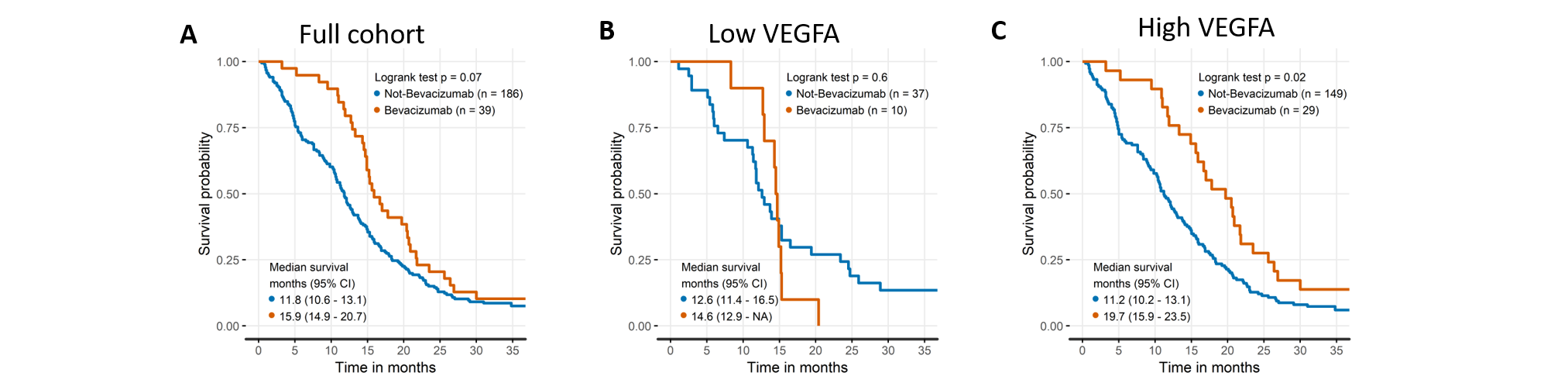
 Additional file: Figure S11.** Kaplan-Meier overall survival (OS) curves for GBM patients treated and non-treated with bevacizumab. TCGA IDH-wt cohort was used to make the analysis.
